# Human Dental Pulp Stem Cells Display a Potential for Modeling Alzheimer Disease-Related Tau Modifications

**DOI:** 10.3389/fneur.2020.612657

**Published:** 2021-01-25

**Authors:** Karlen Gazarian, Luis Ramirez-Garcia, Luis Tapía Orozco, José Luna-Muñoz, Mar Pacheco-Herrero

**Affiliations:** ^1^Laboratorio de Reprogramación Celular, Departamento de Medicina Genómica y Toxicología Ambiental, Instituto de Investigaciones Biomédicas, Universidad Nacional Autónoma de México (UNAM), Ciudad de México, Mexico; ^2^National Dementia BioBank, Ciencias Biológicas, Facultad de Estudios Superiores, Cuautitlán, Universidad Nacional Autónoma de México (UNAM), Cuautitlán Izcalli, Mexico; ^3^Banco Nacional de Cerebros-UNPHU, Universidad Nacional Pedro Henríquez Ureña, Santo Domingo, Dominican Republic; ^4^Neuroscience Research Laboratory, Faculty of Health Sciences, Pontificia Universidad Católica Madre y Maestra, Santiago De Los Caballeros, Dominican Republic

**Keywords:** tau protein, phosphoepitope, Alzheimer disease models, dental pulp cells, neurodegeneration, tauopathies

## Abstract

We present here the first description of tau in human dental pulp stem cells (DPSCs) evidenced by RT-PCR data on expression of the gene MAPT and by immunocytochemical detection of epitopes by 12 anti-tau antibodies. The tau specificity of eight of these antibodies was confirmed by their affinity to neurofibrillary tangles (NFTs) in Alzheimer's disease (AD) postmortem brain samples. We therefore used DPSCs and AD brain samples as a test system for determining the probability of the involvement of tau epitopes in the mechanisms converting tau into NFT in AD. Three antibodies to non-phosphorylated and seven antibodies to phosphorylated epitopes bound tau in both DPSCs and AD NFTs, thus suggesting that their function was not influenced by inducers of formation of NFTs in the AD brain. In contrast, AT100, which recognizes a hyperphosphorylated epitope, did not detect it in the cytoplasm of DPSCs but detected it in AD brain NFTs, demonstrating its AD diagnostic potential. This indicated that the phosphorylation/conformational events required for the creation of this epitope do not occur in normal cytoplasm and are a part of the mechanism (s) leading to NFT in AD brain. TG3 bound tau in the cytoplasm and in mitotic chromosomes but did not find it in nuclei. Collectively, these observations characterize DPSCs as a novel tau-harboring neuronal lineage long-term propagable *in vitro* cellular system for the normal conformational state of tau sites, detectable by antibodies, with their state in AD NFTs revealing those involved in the pathological processes converting tau into NFTs in the course of AD. With this information, one can model the interaction of tau with inducers and inhibitors of hyperphosphorylation toward NFT-like aggregates to search for drug candidates. Additionally, the clonogenicity of DPSCs provides the option for generation of cell lineages with CRISPR-mutagenized genes of familial AD modeling.

## Introduction

Alzheimer's disease (AD), characterized by neuronal cell death, is the most widespread and harmful neurodegenerative disease in the world, and it accounts for more than a half of all cases of dementia (1, 2). Despite significant progress in the understanding of this ailment, the neurobiological mechanisms underlying AD are unknown, and no successful cure has been identified (3, 4). *In vivo* and *in vitro* experimental models have been used to study the pathophysiology and explore therapeutic candidates for this disease (5–7). Brain banks have been of utmost importance for a better understanding of AD. Tau protein, a microtubule-associated protein, was discovered over four decades ago (8). Tau is encoded by a single-copy gene (MAPT) with 16 exons located on chromosome 17q21.3 (9, 10). The alternative splicing of MAPT generates six isoforms of tau in the central nervous system (CNS) [(11–17), reviewed in (18)]. In healthy individuals, tau is distributed nonrandomly in the brain (19) with predominant localization to axons (20), dendrites (21), and synapses (22). Tau has also been evidenced in oligodendrocytes (23), in astrocytes (24), and in some nonneural cells, such as skin fibroblasts (25). Tau function and pathogenesis are restricted to the cytoplasm; hence, the meaning of its presence in nuclei (26–28) remains generally understudied. Tau abnormalities are commonly observed in many neurodegenerative diseases including AD, Parkinson's disease, and Pick's disease. A growing amount of evidence suggests that Aβ oligomers in concert with hyperphosphorylated tau (pTau) serve as the major pathogenic drivers of neurodegeneration in AD. The functions of tau are regulated by its phosphorylation on specific serine/threonine and a few tyrosine residues, which disrupts tau association with microtubules in a physiologically normal manner (14). Under pathological conditions, tau is abnormally hyperphosphorylated (29, 30), introducing into tau a molecule with up to fourfold more phosphates (6–8 mol/mol of tau) than those contained in a monomer (~1.9 mol/mol of tau) (31). Excessively phosphorylated tau monomers lose their affinity for microtubules, self-assembling to form insoluble aggregations or paired helical filaments (PHFs), which are the precursors of neurofibrillary tangles (NFTs) (32, 33) considered a key marker of AD (34). Phosphorylation of tau results in microtubule instability, ineffective transport of molecules and organelles, and incompatibility with neuronal function.

Tau is the predominant constituent of NFTs (35, 36). Antibodies against tau and NFTs (37–39) have allowed NFT characterization in AD pathogenesis (40). However, the process underlying the formation of NFTs is unknown. In normal brains, tau can undergo transient aggregations such as oligomeric structures in the cortex (41, 42) and more advanced PHFs in neurons of hibernating animals (43–45) and fetal brains (46, 47), suggesting the existence of a physiological mechanism of *reversible* aggregation of tau. However, these observations could not be reproduced *in vitro*. The cell models used so far for tau studies are either malignant [neuroblastoma, PC12; (48)] or transgenic (49, 50) cell lines suffering from the consequences of genomic modifications in the epigenetic state different from normal cells with endogenous tau protein.

In the present study, we describe for the first time the presence of an endogenous tau protein in human dental pulp stem cells (DPSCs) evidenced by the detection of its non-phosphorylated and phosphorylated epitopes by a panel of tau-specific antibodies, some of which are used for characterization of tau modifications in the AD. With this discovery and their previously described capacity to develop into neurons in other (51, 52) and our (53) studies, DPSCs demonstrate their potential of modeling tau normal state and possibility of inducing changes mimicking the modifications occurring during neurodegeneration. The principal advantage of DPSCs is their being authentic neuronal cell progenitors. Other tissue origin multipotent stem cells, such as bone marrow (54), adipose tissue (55), umbilical cord blood (56), and spleen and thymus (57), have been shown to develop into cells with neuronal phenotype. However, these cells, collectively defined as “mesenchymal stem cells” [a term coined by Arnold Caplan in 1991 (58)] and approved for extensive use by the International Society for Cellular Therapy position statement (59), have two crucial differences from dental cells, which were discussed in our previous publications (53, 60): (i) they originate from mesenchyme that is developmentally unrelated to the nervous system, and (ii) none of them has been shown to contain tau protein because mesenchyme origin cells do not express this protein *in vivo*. The phenomenon of their conversion *in vitro* into cells with neuronal phenotype might result from the epigenetic plasticity that stem cells possess. As neurons produced from these stem cells lacked tau protein, they can be of utility in regenerative medicine but not in modeling tau-related aspects of neurodegeneration. In contrast, DPSCs are known from embryological (61) studies to stem from ectodermal–neuroepithelial–neural crest lineage producing, among many cell types, cells of the peripheral neural system and glia (62, 63).

Currently, apart from the neurons produced from *pluripotent* stem cells able to model AD (7, 64), neural crest origin *multipotent* stem cells, with DPSCs as the most known representatives, represent the most relevant and easy-to-use cellular system capable of recapitulating *in vitro* the tau aggregation toward NFT-like pathogenicity. This novel experimental strategy, combined with existing *in vitro* AD modeling approaches, can contribute to a better understanding of pathological mechanisms underlying AD and the development of effective therapeutics.

## Materials and Methods

### Isolation and Culture of DPSCs

Deciduous teeth were collected from 7- to 8-year-old male children in a dental clinic in Mexico City. Informed consent was obtained from their parents. The study was approved by the Bioethics Committee of the Biomedical Research Institute at the National Autonomous University of Mexico. The lower primary front teeth that usually erupt first and thus also the first to fall were readily eliminated by odontologists without any surgical procedure and inspected, and those with any visible or suspected abnormality were placed in sterile Hank's balanced salt solution (Gibco, Thermo Fisher Scientific, Waltham, MA) with 2X antibiotic–antimycotic solution (Anti-Anti; Gibco), transferred in a special transport to the laboratory and processed under sterile conditions within 24 h as previously described (60). Briefly, teeth in the laboratory were repeatedly washed with commercial mouthwash solution (Listerine Cool Mint, Johnson and Johnson, New Brunswick, NJ) and then with 2X Anti-Anti in phosphate-buffered saline (PBS; Gibco). Teeth were mechanically broken with a pincer to expose the pulp, which was minced in a sterile glass Petri dish and digested using a 3 mg/ml solution of collagenase type I (Sigma-Aldrich, St. Louis, MO) in PBS for 60 min at 37°C. Enzymes were inactivated by diluting with Dulbecco's Modified Eagle Medium (DMEM) and Ham's F-12 medium (1:1 ratio, Gibco) supplemented with 10–20% fetal bovine serum (FBS, Gibco), in a 5% CO_2_ environment, until small colonies of spindle-shaped cells appeared. Colonies and single cells were removed from the dish by 5-min digestion with TrypLE Express (Invitrogen, Thermo Fisher Scientific), seeded in T25 bottles, and cultured during no more than five passages before being used. The ability of DPSCs to respond to inducers of the neuronal lineage was tested, as described (53). During this period, healthy tooth bone preserves the normal state of the pulp to be used and the reproducibility of the cells isolated from them and cultured. In our studies, healthy tooth bone preserved the viability of the pulp for at least 30 h as evidenced by the preservation of the proliferation, phenotypic, and stemness characteristics of the obtained and cultured cells during at least five passages of cell populations.

### Phase Contrast Microscopy With Immunofluorescence Equipment

An IX71 phase contrast microscope (Olympus, Tokyo, Japan) and QCapture Suite software (QImaging, Surrey, BC) were used for image analysis. A confocal microscope, SP8 (Leica, Wetzlar, Germany), was used for immunocytochemical analyses.

### Antibodies

Supplementary Table 1 presents the panel of the 12 antibodies used in this study, indicating the tau protein amino acids implicated in the three types of epitopes that induced them: non-phosphorylated, phosphorylated, and hyperphosphorylated. The antibodies were of the class IgG, except for TG3 (IgM). The tau specificity of the antibodies has been confirmed in earlier (65) and recent (28) studies. The antibody to glycogen synthase kinase 3β (GSK3β) was from EMD Millipore Billerica (MA, USA). Human β-amyloid-specific monoclonal antibody (BAM-10) was from Invitrogen (Thermo Fisher Scientific cat. #MA1-91209). Secondary antibodies used were anti-mouse and anti-rabbit IgGs (Fc mouse and Fc rabbit; see Supplementary Table 1).

### Immunofluorescence Assays of DPSCs

DPSCs were plated in glass chamber slides and cultured in two passages. The cells were then fixed with 4% paraformaldehyde in PBS for 30 min at room temperature (RT) and permeabilized with 0.1% Triton X-100 (Sigma-Aldrich) in PBS for 10 min at RT. Nonspecific binding was blocked with 1% bovine serum albumin (BSA; MP Biomedicals, Santa Ana, CA) in PBS for 2 h at RT. DPSCs were incubated with the primary antibodies diluted in blocking solution (1% BSA in PBS) overnight at 4°C and then with one of these secondary antibodies for 1 h at RT in the dark: (1) Alexa 488-tagged secondary antibody, (2) fluorescein isothiocyanate-tagged goat anti-rabbit immunoglobulin G, (3) tetramethylrhodamine-tagged goat anti-mouse immunoglobulin G, or (4) cyanine 5-tagged goat anti-mouse IgM. Nuclear counterstaining was done with TOP-RO-3 (Thermo Fisher Scientific) or Hoechst. Rhodamine phalloidin was used for the tetramethylrhodamine-conjugated filamentous actin staining.

### Origin and Immunofluorescence of AD Tissue Sections

**Figure 1:**
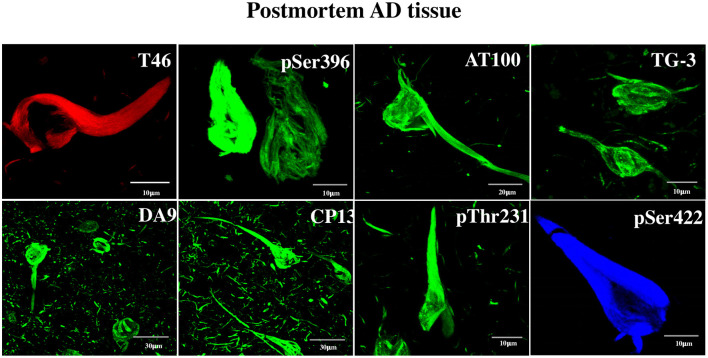
Detection by anti-tau antibodies of NFTs formed in AD brain. Immunofluorescent data showing the affinity of the eight antibodies to postmortem AD brain NFTs, which served as tools for the discovery of tau in DPSCs. The specificity of the antibodies is as follows. DA9, T46: recognize epitopes not requiring phosphorylation; CP13 (aa T231), pT231, pS396, pS422: recognize moderately phosphorylated epitopes; TG3, AT100: recognize hyperphosphorylated epitopes forming NFTs. Channels: red (T46), blue (pS422), green (the rest). For information on the critical amino acids of the tau epitopes recognized by these antibodies, see Materials and Methods.

AD human tissue was obtained from the National Dementia Biobank, Mexico, in accordance with the institutional bioethical guidelines. A representative immunoassay image confirming the affinity of the eight antibodies for AD NFTs (Figure 1) was from the studies of the Laboratory of Diagnosis and Investigation of the Mexican National Dementia BioBank (Facultad de Estudios Superiores, Cuautitlán, UNAM, Estado de México, México) (postmortem brains of Braak stages 5 and 6, used at PM 5–8 h, of four females aged 70, 70, 75, 80 years and two males aged 78 and 89 years with AD). Histological sections were prepared, blocked with 0.2% IgG-free albumin (Sigma Chemical Co.) in PBS for 20 min at RT, and then incubated with the primary antibody overnight at 4°C. Tissue slices were then incubated with the secondary antibodies: FITC-tagged goat–anti-mouse IgM or FITC/TRITC-tagged goat–anti-mouse IgG or FITC/CY5-tagged goat–anti-rabbit IgG secondary antibody (Jackson ImmunoResearch Laboratories, Inc., West Grove).

For confocal microscopy analysis, immunolabeled AD brain sections of the hippocampus were mounted in an anti-quenching media, VECTASHIELD (Vector Labs, Burlingame), and examined with a confocal laser scanning microscope (TCP-SP8, Leica, Heidelberg), using a 100X oil-immersion plan apochromat objective (NA 1.4). Ten to 15 consecutive single sections were sequentially scanned at 0.8- to 1.0-μm intervals for two channels throughout the z-axis of the sample. The resulting stack of images was projected and analyzed onto the two-dimensional plane using a pseudocolor display of green (FITC), red (TRITC), and blue (CY5). Fluorochromes were excited at 488 nm (for FITC), 530 nm (for TRITC), and 650 nm (for CY5).

### RT-PCR Assay

Total RNA was isolated using TRIzol reagent following the manufacturer's instructions (Invitrogen). RNA isolation was followed by DNase I treatment (Invitrogen). RNA was cleaned up using the RNeasy mini kit (Qiagen). Reverse transcription and DNA amplification were performed using the One-Step RT-PCR kit (Qiagen). The PCR primers used were as follows:

GAPDH forward: 5′AAGGTGAAGGTCGGAGTCAA;

GAPDH reverse: 5′AATGAAGGGGTCATTGATGG;

MAPT forward: 5′CCAAGTGTGGCTCATTAGGCA;

MAPT reverse: 5′CCAATCTTCGACTGGACTCTGT;

CD44 forward: 5′CTGCCGCTTTGCAGGTGTA; and

CD44 reverse: 5′CATTGTGGGCAAGGTGCTATT.

The reliability of the described results was ensured by the repetitions of the assays and presentation of representatives.

## Results

### Testing the Specificity of Tau Antibodies in NFTs in the AD Brain

Non-phosphorylated and phosphorylated tau immunoreactivity in NFTs from the AD brain was tested. It was done with two purposes: (a) to serve as a positive control for the tau immunochemical assay in DPSCs and (b) to obtain information about the antigenically functional state of the tau epitopes in NFTs. This shows clearly that each of the tested antibody recognized its epitope in NFTs, evidencing its antigenically active state (Figure 1). The antibodies have been used previously in studies of AD at the National Dementia BioBank (Ciencias Biológicas, Facultad de Estudios Superiores, Cuautitlán, UNAM, Estado de México, México; Banco Nacional de Cerebros-UNPHU, Universidad Nacional Pedro Henríquez Ureña; and Neuroscience Research Laboratory, Faculty of Health Sciences, Pontifica Universidad Católica Madre y Maestra Santiago de los Caballeros, República Dominicana).

### DPSCs Harbor mRNA of Tau MAPT Gene and Tau Protein, Which Are Phosphorylated by GSK3β and Aβ Precursor Protein (APP)

**Figure 2:**
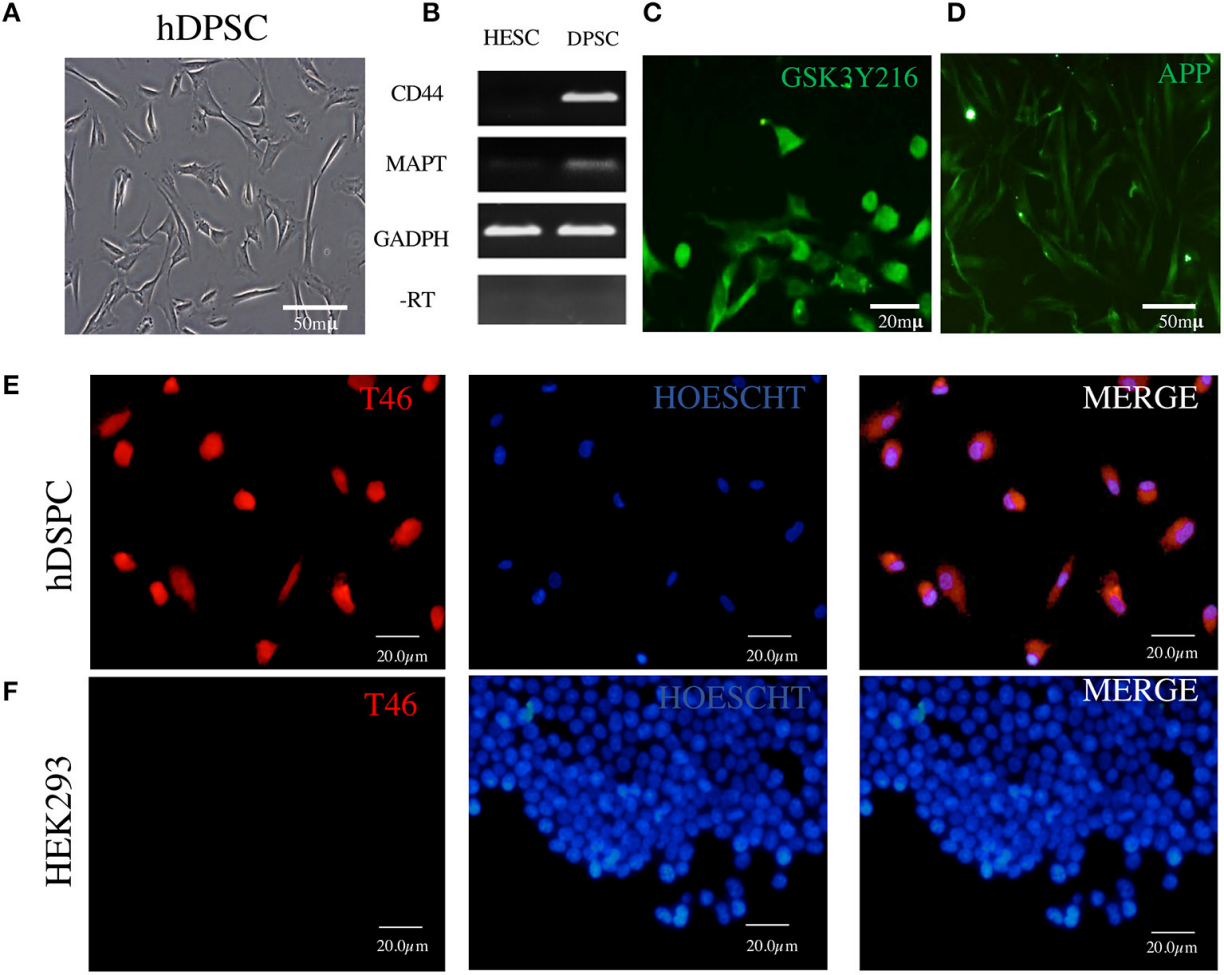
Detection in DPSCs of tau RNA, tau protein, GSK3β, and APP. **(A)** Fibroblast-like morphology of DPSCs in fetal serum-containing medium. **(B)** RT-PCR assay showing cDNA copies of tau MAPT and CD44 RNAs; RNA of human embryonic stem cells (ESCs) used as a negative control and RNA of GAPDH as a positive control. **(C,D)** Immunofluorescence of GSK3β and APP. **(E)** Immunofluorescence of tau protein revealed by T46 antibody in DPSCs. **(F)** HEK293 cells used as a negative control for T46 specificity. The nuclei are stained with Hoechst.

**Figure 3:**
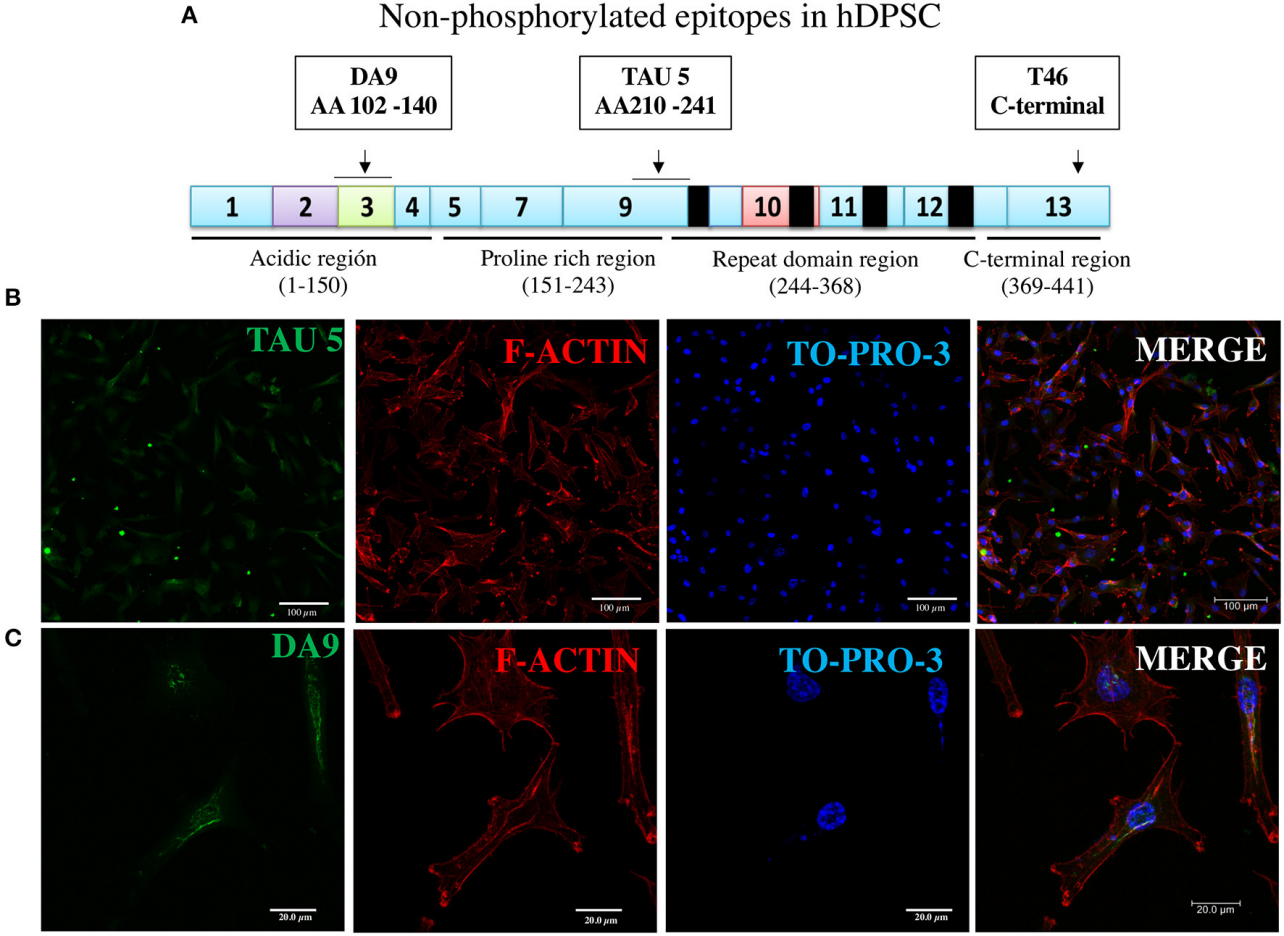
Immunofluorescence of two additional non-phosphorylated tau epitopes. **(A)** Epitopes of TAU5 and DA9 antibodies reveal their non-phosphorylated epitopes localized to N-terminal exon 3 and proline-rich exon 9 regions, respectively. (**B,C**) Immunofluorescent staining of the tau revealed in DPSCs by the antibodies. Nucleic acids and F-actin are counterstained with TO-PRO-3 and rhodamine phalloidin, respectively.

**Figure 4:**
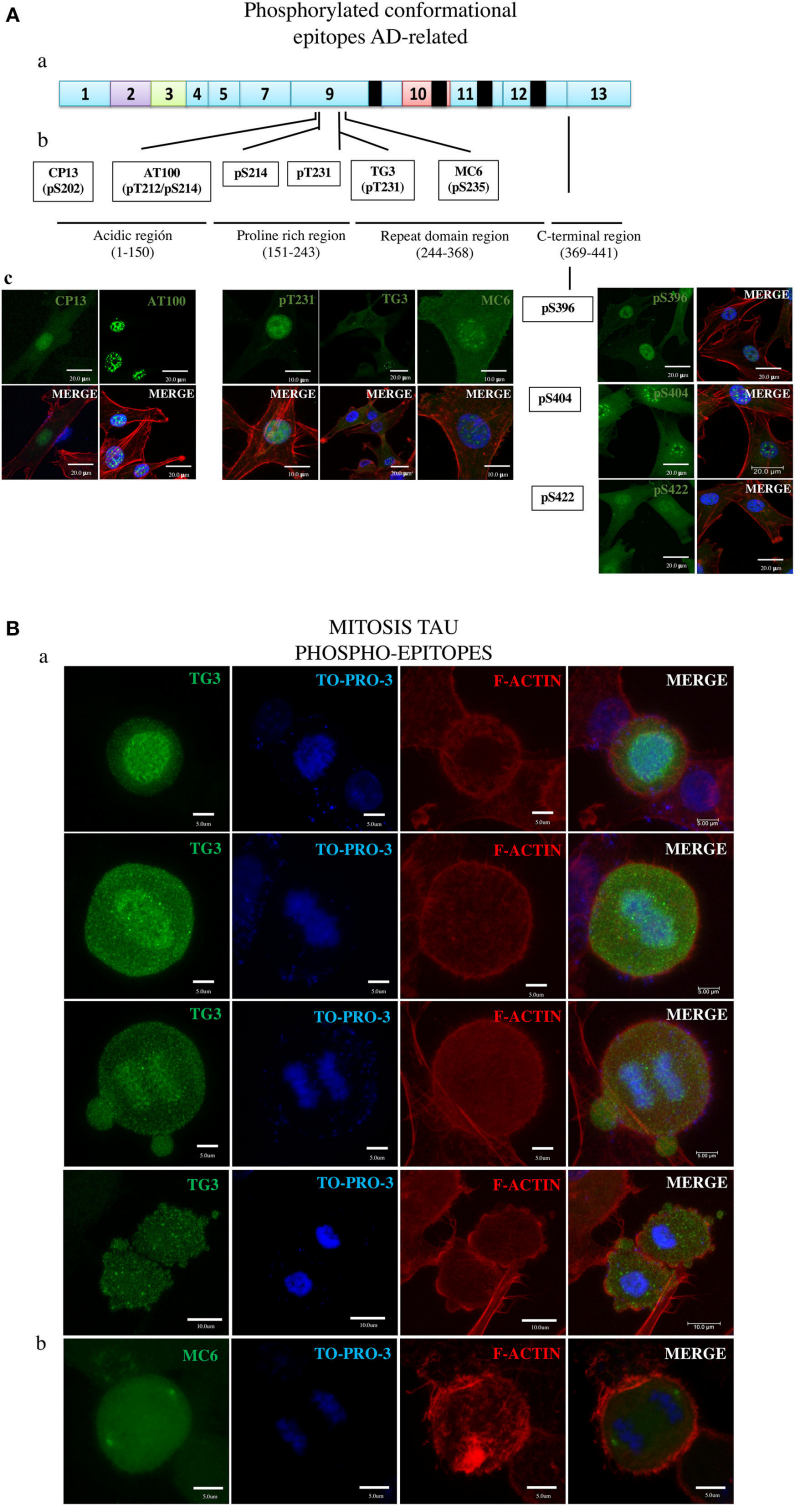
Overall presentation of the tau protein fluorescence revealed in DPSCs by antibodies to phosphorylated epitopes. **(A)** Tau in interphase cells. (ab) Localization of epitopes to tau protein proline-rich exon 9 and C-terminal exon 13 sites. (b) Amino acids involved in the epitopes of the moderately phosphorylated proline-rich (CP13, pS214, pT231, MC6, and pS369) and C-terminal (pS369, pS404, and pS422) epitopes. (c) Imunofluorescence images. **(B)** Tau in mitosis. (a) TG3 reveals tau associated with chromosomes at consecutives phases of mitosis. Counterstaining with TO-PRO-3 and rhodamine phalloidin. (b) MC6 bound to its epitope in the tau associated with centrosomes. (c) Immunofluorescent staining of tau by means of the antibodies.

The major difficulty in the studies of tau in AD brain is that the NFTs represent the advanced stages of the tau aggregation process in the environment of neuronal degeneration. Therefore, neural-lineage cellular systems capable of recapitulating the earlier stages of tau aggregations are needed. Dental stem cells have been shown by several groups to differentiate into cells with neuronal phenotype; however, the major marker of neuronal identity, tau, has not been shown to exist in those neurons (51–53). Therefore, we have undertaken assays to see whether the cells contain endogenous tau detectable by RT-PCR and anti-tau antibodies. Figure 2A shows DPSCs cultured in growth-promoting medium supplemented with high (10%) FBS (see Materials and Methods). Under this condition, the cells display properties characteristic of the class of mesenchymal stem cells (59): fibroblast-like morphology; plastic adherence; the markers CD73, CD105, and CD90; and tri-lineage (bone-oriented) differentiation potential (53, 60). We recently reported on the ability of these cells to display under serum-less culture conditions an upregulated Wnt/β-catenin signaling neural crest features (CD271/p75, CD57/HNK1, Sox10, among others), associated with the neural-lineage fate which is evidenced by the expression of neural markers and production of neurotropic factors (see Supplementary Figure 5) and the capacity of responding to a neurogenic environment via the transient expression of the neuronal commitment gene Sox2 and then neural marker beta tubulin (53). To complement these neuronal properties of DPSCs, we show here the presence of tau in these cells detected at the gene expression and protein levels. An RT-PCR assay (Figure 2B) revealed the RNA copies of MAPT gene known to encode the amino acid sequences of the six tau isoforms in the brain of AD persons (66, 67). The tau protein was immunochemically detected (Figures 2–4 and Supplementary Figures 1–4) by means of 12 antibodies (Supplementary Table 1), of which the T231 revealed it as threads seemingly associated with microtubules (Supplementary Figure 1).

Collectively, these results underscore the tau in DPSCs as unique neural components involved in normal and pathological cellular processes. Below, we describe in detail the results obtained in these immunocytochemical analysis.

In the immunocytochemical experiments, apart from the staining of tau with antibody, we used counterstaining of nuclei and cytoplasmic filamentous actin with TO-PRO-3 and rhodamine phalloidin, respectively, confirming cellular integrity. In the first round of fluorescence immunostaining experiments, we used three antibodies to non-phosphorylated epitopes localized to exons of three different protein sites (Supplementary Table 1).

The assay with antibody T46 (which bound a tangle of AD brain, see Figure 1) identified an unphosphorylated epitope in the tau protein as a homogeneously stained component of the cytoplasm and of the nucleus of majority of the cells (Figure 2E and Supplementary Figure 2C). The specificity of the immunochemical reactions was proven by the *positiveness* of the T46 to tau in AD brain NFTs (Figure 1) and the *negativeness* to the irrelevant HEK293 (Figure 2F). This result indicated the presence of the immunogenically active state of the T46 epitope in both these tau-containing cells (DPSCs and AD brain). The two other antibodies to non-phosphorylated epitopes, Tau5 (involving exon 9 aa 210 to aa 241 residues) and DA9 (recognizing exon 4 and 5 aa 102 to aa 140 residues) (see Supplementary Table 1), showed a similarity to the T46 tau protein homogeneously arranged in the cells (Figure 3 and Supplementary Figure 2). The detection of these three epitopes in N-terminal exon 3 (DA9), central (exon 9), and C-terminal end (exon 13) regions of the protein, along with the expression of the MAPT gene (Figure 2B), suggested the presence in DPSCs of the full-length tau protein containing six isoforms previously documented for tau in AD brain (67). The detection in these experiments of tau in nuclei of DPSCs confirmed the previous data (27) but did not add novel information elucidating the role of this tau. The presence in DPSCs of the GSK3β, known to phosphorylate tau (68), and the APP, the source of toxic amyloid peptides, enhancing this kinase activity to the level of hyperphosphorylation and formation of NFTs in AD (69–71).

### Moderately Phosphorylated Tau Epitopes Were Active Both in DPSCs and in Postmortem AD Brain

Seven phosphorylated epitopes formed two groups, one consisting of three epitopes (pS396, pS404, and pS42; Figure 4A, right side) localized to the C-terminal exon 13 and the other one including the epitopes pT231, pS202 (CP13), and pS235 (MC6) localized to the proline-rich exon 9 site. Of these, the pS396, pS422, pT231, and CP13 showed the ability to detect AD NFTs (Figure 1). In DPSCs, the pS396 antibody predominantly bound nuclear tau and only barely the cytoplasmic tau. The immunostaining seen for pS404 and pS422 antibodies revealed a very intense signal in the cytoplasm and in the nuclei with the difference that nuclear tau revealed by pS404 formed approximately 10 small compact bodies per cell, compared to a diffuse distribution of nuclear tau revealed by pS422. Antibodies of the epitopes pT231, pS202 (CP13), and pS235 (MC6) recognized their phosphorylated epitopes in DPSCs via a homogeneous staining of the cytoplasm and the nuclei (Figure 4 and Supplementary Materials).

### AT100 and TG3 Distinguished Between the Cytoplasmic and Nuclear Tau but in a Reciprocal Manner

The results of testing AT100 and TG3 (Figure 4A and Supplementary Figures 3A–C) are of great interest due to their being induced by AD NFTs (38). Consistent with this origin, the AT100 epitope in NFT is considered a hallmark of AD (34), and the antibody is exploited as the most specific reagent in AD immunodiagnostics (37). Consistent with this known feature, the AT100 in this study did not bind to the cytoplasmic tau of DPSCs (Figure 4A and Supplementary Figure 3A), confirming the absence of its antigenically active epitope in these normal cells. In contrast, TG3 was positive for the cytoplasmic and negative for the nuclear tau (Figure 4A and Supplementary Figures 3B,C).

### Tau in Mitotic Cells

Remarkably, the TG3 antibody was the only one in the panel that labeled tau in mitotic chromosomes at all phases of the mitosis (Figure 4A) in agreement with the observation by others (28, 38, 72). We found rare cells at the anaphase–telophase phases showing low tau amounts in recently reformed nuclei (Supplementary Figure 3C), which, along with the above shown presence of tau in mitotic chromosomes, suggests that tau appears in mitosis and disappears in the nuclei of interphase cells. Figure 4C and Supplementary Figure 4 present the tau in centrosomes revealed by MC6.

## Discussion

### DPSCs Are Ectodermal Neural Crest Origin Tau-Containing Neuronal Precursors

Cellular models able to reproduce different facets of AD pathogenesis are needed to support continued efforts to learn about the disease. *In vivo* processes in neurons are not accessible in live brains, and rare biopsy samples often die in cultures. Majority of the studies of *in vivo* neurodegeneration are done using biopsy material from fixed postmortem brains containing the advanced stages of tau modifications. It is possible to reconstruct the initial steps of pathogenesis via a meta-analysis of independently obtained experimental data, but such reconstructions may not be reliable. Neuronal precursors have been obtained from human induced pluripotent stem cells (51, 52), including cells with mutations that cause familial AD (73) or cells that have been genetically manipulated toward AD pathogenesis (74). The potential differentiation of these cells into neurons is the advanced strategy for recapitulating amyloid β and tau pathology in advanced human neural cell culture models of AD (71). This strategy has recently been improved through the use of 3-D AD models, which have emerged as an advanced alternative to 2-D models (73, 75). The drawbacks of the currently used 3-D models of AD include insufficient maturation and their inaccessibility for routine employment.

The task of these and other novel models is to display *in vitro* the tau modification and to collect novel experimental observations regarding the current view that the hyperphosphorylation-induced tau aggregations and formation of the NFTs are associated with neurodegeneration and that abnormal processing of APP and accumulation of β-amyloid triggers this process in a combination with stress factors as a step toward preclinical drug discovery, which holds potential for personalized therapeutic applications.

DPSCs widely known as mesenchymal cells, now shown to contain tau, have a potential to be a novel easier-to-use cellular system for modeling tau-mediated pathogenesis. The potential relies substantially on the recently demonstrated neural crest identity treats (epithelial morphology and a set of specific markers such as P75, HNK1, and Sox10 displayed in experiments) (60) on their culture in media required for embryonic neural crest cells produced *in vitro* from pluripotent stem cells (76). Under appropriate neurogenesis induction culture conditions, DPSCs develop into cells with neuronal phenotype including axon and dendrite-like outgrowths (51, 52). Nevertheless, since DPSCs do not participate in the innervation of teeth, their neuronal identity was unclear. This study presents two novel features of DPSCs essential for the validation of the neurons they produce and the capacity of these neurons to model tau implication in neurodegeneration: (i) the description of tau provided direct proof for their neural identity, and (ii) the display of the epitopes recognized by a panel of anti-tau antibodies permits the investigation of tau modifications. A conceivable explanation for these neural properties of DPSCs is that the cells are endowed by neural crest with epigenetic plasticity, permitting the reprogramming of dental pulp cells to neural crest stage cells (77) and proceeding to other lineages (78), here to neural lineage of the peripheral system that, as in the DPSC population, is the derivative of the neural crest (79). With these novel characteristics, DPSCs can be used in studies of AD and other tauopathies to compare the functional states of tau in these physiologically normal cells and in NFTs available from postmortem AD brains.

### DPSCs as a Novel System for Modeling Normal and Pathological Tau Modifications

This study aimed to examine the properties of tau in DPSCs as a prospective system for modeling the initiation of tauopathy. The findings describe the functional state of the epitopes recognized by 12 antibodies that were frequently employed in studies of tau aggregated forms in postmortem brains of AD patients (see Introduction). First, we obtained reliable data on the presence of tau in these cells at RNA and protein levels; furthermore, it is suggested that the full-length protein is produced considering the detection of tau epitopes in the N-terminal, C-terminal, and central, proline -rich domains containing the residues responsible for the interaction with microtubules. The counterstaining with phalloidin reveals in multichannel immunofluorescent merged images of tau positioning along phalloidin-revealed threads, presumably microtubules. Based on this finding, the prospective aim was to use tau in two types of studies: (a) to test comparatively the functional state of its epitopes in monomer tau of DPSCs and in NFTs of the AD brain samples and (b) to test the propensity of the protein sites in which these epitopes are located to undergo initial steps of aggregation toward NFTs under the influence of currently known *in vitro* inducers of tau phosphorylation aggregation to model the formation of NFT, such as okadaic acid (79, 80) and inhibitors of these processes as drug candidates against neurodegeneration (81). In this study, we describe the results of the first study hypothesizing that the active or inactive state of epitopes (evaluated by the binding/nonbinding of the respective antibody) in monomer tau of DPSCs and in its aggregated NFT form in samples from AD brains would provide information about the mode of the participation of respective tau sites in neurodegeneration. The active state of epitopes in DPSCs and in AD indicated that the phosphorylation and conformational events that activated them were not induced by neurodegeneration. In contrast, the active state of epitope in AD NFTs but not in DPSCs indicated that the epitope was activated by neurodegeneration factors. Based on these assumptions, the study compared the ability of binding of 12 antibodies to their epitopes in DPSCs (normal monomer tau) and AD (pathological NFT) brain samples.

Three antibodies (Dau9, Tau5, T46), whose epitopes do not need phosphorylation, (i.e., are permanently active), were used to standardize the conditions of immunocytochemical assays of other epitopes. These antibodies bound tau in both DPSC (Figures 2, 3 and Supplementary Materials) and AD brain sample (Figure 1); therefore, the tau sites of their locations were beyond the processes converting monomer tau into NTF. The activity of nine antibodies was dependent on phosphorylation. Six out of the nine antibodies (CP13, pS214, T231 in the protein central “proline rich” exon 9 region spanning aa151 to aa243, and the pS396, pS404, pS422 located in the C-terminal tau aa369–aa441 region; see Figure 4A) bound their epitopes in both the normal (monomer) tau in DPSCs and four of them showed their affinity for pathological (aggregated) tau in AD NFTs (Figure 1). The results suggested that these phosphorylated epitopes, like the three unphosphorylated epitopes, were phosphorylated and immunogenically active in monomer tau and remained in this state during tau aggregation to form NTFs. The representing these epitopes tau residues (from N- to C-terminal direction: Ser202, Ser214, Thr231, Ser396, Ser404, and Ser422.) are therefore considered by this study to not depend on the process of tau pathological aggregation and formation of NFTs in AD. Antibodies of the two remaining epitopes, AT100 and TG3, showed results that differed from those described above, and displayed properties compatible with the ability of DPSCs to model their relation to AD. Previous studies of these two epitopes have suggested two properties that are relevant to the role of tau in AD. First, these epitopes were generated by immunizing mice with NTFs purified from postmortem AD brains (38) that possessed tau-specific antigenicity (35, 36, 82). Second, the establishment immunogenically active form of these epitopes required preliminary phosphorylation of other epitopes. Specifically, the functionally active state of AT100 epitope depends on sequential phosphorylation of the AT8 epitope residues Ser202 and Thr205, followed by phosphorylation of Thr212 and Ser214, both of which are critical for recognition by AT100 of its epitope (83, 84). Each of these phosphorylation steps was strongly required to prime the phosphorylation of the following it residue; for example, if Ser214 would be abnormally phosphorylated first, the phosphorylation of the preceding residues would be blocked and the epitope would not be active in the AD-related state (83). Existing evidence assumes this priming process of the epitope activation to occur only when neurodegeneration factors induce formation of NFTs thus considering this epitope in NFT as a hallmark of AD and the AT100 antibody as its most specific diagnostic. The fact that AT100 did not bind tau in the cytoplasm of DPSCs but bound AD NFTs provides an independent proof for the diagnostic potential of AT100 antibody. Unexpectedly, the same AT100 had a strong reaction with nuclear tau; however, the origin and the structural state of tau in nuclei, and its relation to tau pathogenesis remain to be elucidated (27).

The active state of the TG3 epitope requires phosphorylation of threonine 231 (see Supplementary Table 1 and Figure 4) by GSK3β, which has been shown to depend on prior (priming) phosphorylation of T235 by CDK5 (85, 86). Importantly, phosphorylation of T231 is vigorously required for the assembly of tubulin into microtubules, and, together with the S214, the T231 is known as a tau phosphorylation site (87) crucial for the Tau–MT interaction (87, 88). Interestingly, tau revealed by pt231 in DPSCs looks as if it co-localizes putative microtubules (Supplementary Materials, Figure 1). The most likely explanation for the above-described findings is as follows. The differential immunogenic activation of the epitopes of AT100 and TG3 antibodies in nuclear (AT100), cytoplasmic, and mitotic chromosome-associated (TG3) tau seems to be regulated by the priming mechanism, which is dependent on the peculiar structural contexts of these cell compartments. In normal cells, the contexts keep these epitopes inactive to protect the cell from their pathogenic potential. Factors inducing neurodegeneration alter the structural contexts which activate the priming system and the respective cell component phosphorylation of these AD-related epitopes. The hypothesis is in an agreement with evidence that AT100 and TG3 are specific to NFTs in the AD brain (38, 72), and according to one scenario (65), they are among the epitopes (pT231 → TG-3 → AT8 → AT100 → Alz-50) sequentially phosphorylated in the course of AD pathogenesis. The inclusion in this sequence of the AT100 and TG3 epitopes is remarkable.

In conclusion, this study describes DPSCs as a novel AD modeling system. The results of this initial study suggest that the epitopes of 10 out of the 12 antibodies we used were active in the monomer tau of DPSCs and passed to NFT independent of the factors that induced this pathological tau form. In contrast, the epitope of AT100 was inactive in DPSCs but active in NFTs, thus demonstrating that the active state of its epitope is not formed in the cytoplasm of normal cells and is formed when the tau is hyperphosphorylated and forms insoluble tangles. Examination of the active state of the TG3 epitope in the cytoplasm of DPSCs represents another possible area for future research. Future experiments will address this issue in a more quantitative manner.

The study findings suggest that DPSCs are suitable for detailed systematic investigations of up to 80 tau serine/threonine phosphorylated sites (87). Each site contributes to the dynamics of the protein conformational pattern, and some of them contain epitopes recognized by antibodies. Their binding to the epitopes in DPSCs and in brain samples at different stages of AD can illuminate the basic mechanisms that regulate tau implication in the disease.

## Data Availability Statement

The original contributions presented in the study are included in the article/Supplementary Material, further inquiries can be directed to the corresponding author/s.

## Ethics Statement

The studies involving human participants were reviewed and approved by Imelda Lopez Villase; Raul Mansilla Jimenez; Agness Odele Fleury. Instituto de Investigaciones Biomédicas, UNAM, Mexico. Written informed consent to participate in this study was provided by the participants' legal guardian/next of kin.

## Author Contributions

KG: conceptualization, project administration, supervision, and writing – original draft. LT: data curation. LR-G, LT, JL-M, and KG: formal analysis. KG and JL-M: funding acquisition and resources. KG, LR-G, and LT: investigation. LT, LR-G, and JL-M: methodology and visualization. LT, LR-G, JL-M, and MP-H: validation. KG, LT, LR-G, MP-H, and JL-M: writing – review & editing. All authors contributed to the article and approved the submitted version.

## Conflict of Interest

The authors declare that the research was conducted in the absence of any commercial or financial relationships that could be construed as a potential conflict of interest.
