# Supplementary material for: Human Dental Pulp Stem Cells Display a Potential for Modeling Alzheimer Disease-Related Tau Modifications
Source: Front Neurol. 2021 Jan 25;11:612657. doi: 10.3389/fneur.2020.612657 (PMC7868559; doi:10.3389/fneur.2020.612657)

Supplementary Material

STable 1 Characteristics of antibodies

Antibody	Class	Epitope
Anti-Tau (T46)	IgG	Non-phosphorylated C-terminal aa?
Anti-Tau (Tau 5)	IgG_1	aa 210-241
Anti-Tau (DA9)	IgG_1	aa102–140
		phosphorylated
Anti-Tau (CP13)	IgG_2	pS202
Anti-Tau (phospho Ser214)	IgG	pS214
Anti-Tau (phospho Thr231)	IgG	pT231
Anti-Tau (TG3)	IgM	pT231
Anti-Tau (MC6)	-	pS235
Anti-Tau (phospho Ser396)	IgG	pS396
Anti-Tau (phospho Ser404)	IgG	pS404
Anti-Tau (phospho Ser422)	IgG	pS422
Anti-Tau (AT100)	IgG	Hyperphosphorylated pT212, pS214
SecondaryAb anti mouse	IgG	Fc mouse
Secondary Ab anti-Rabbit	IgG	Fc rabbit

Supplementary Figures

Magnified images of DPSCs to highlight the details of tau in DPSCs

FigS1. Immunodetection of tau protein in DPSCs.
A. A single cell with tau detected by 231 arranged along the main cellular axis co-localizing to microtubules.
B. Two cells of which one shows concentration of 231-stained tau in the pole that seemingly is moving. F-Actin was highlighted by Falloidin and nucleic acids by TOP-RO3; the figures provides one of the evidences of the presence and functionality of tau protein in DPSCs.
C. A cell with tau and merged with Actin

A **B**

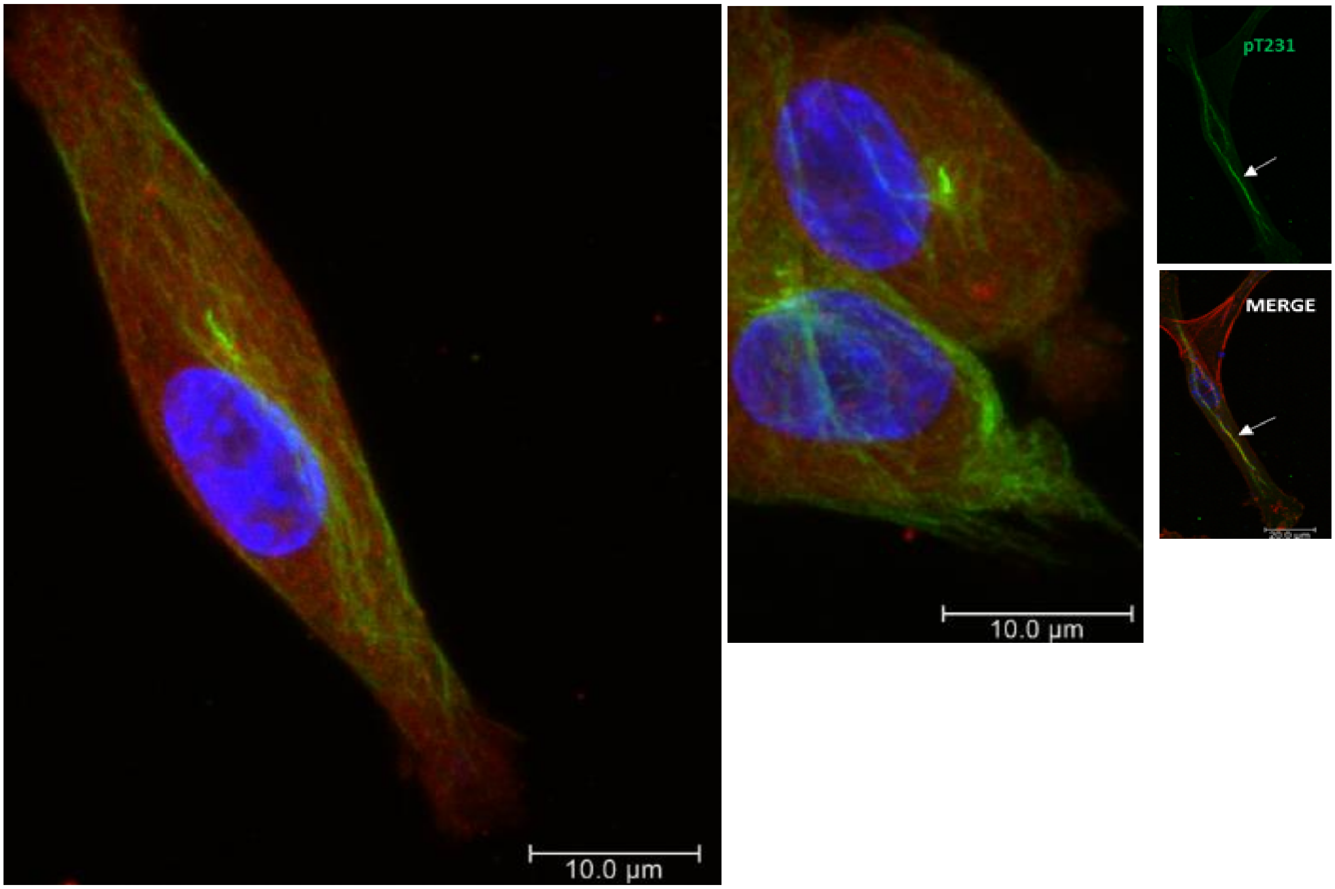

The contrasting properties of AD most specific epitopes of AT100 and TG3

FigS2A

AT100 does not bind the tau in the cytoplasm of DPSCs as an evidence that its epitope is not in active state in these normal cells conforming the diagnostic potential of AT100. The same antibody binds in nuclei to 10-12 compact bodies located in different parts of the chromatin. The structural form and the role(s) of this tau in nuclei is unknown.

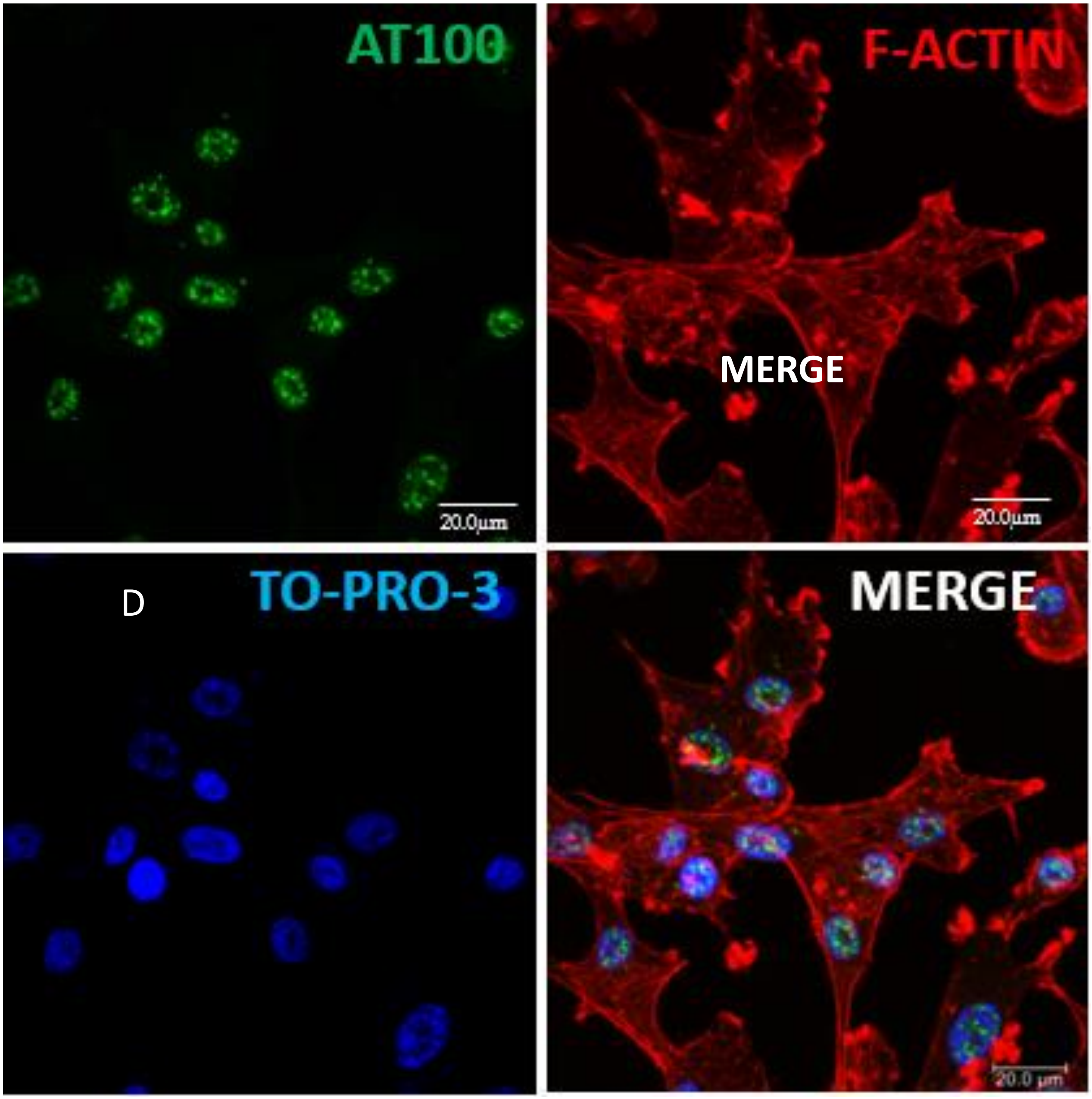

FigS2Ba

TG3 antibody binds reveals tau in the cytoplasm in a "granular form described in AD brain as tau undergoing oligomerization (Maeda et al, 2006; 2008) which can be both a normal state of tau and

the initiation of aggregation. The same antibody fails to bind tau in the nuclei contrasting in this respect with AT100 antibody.

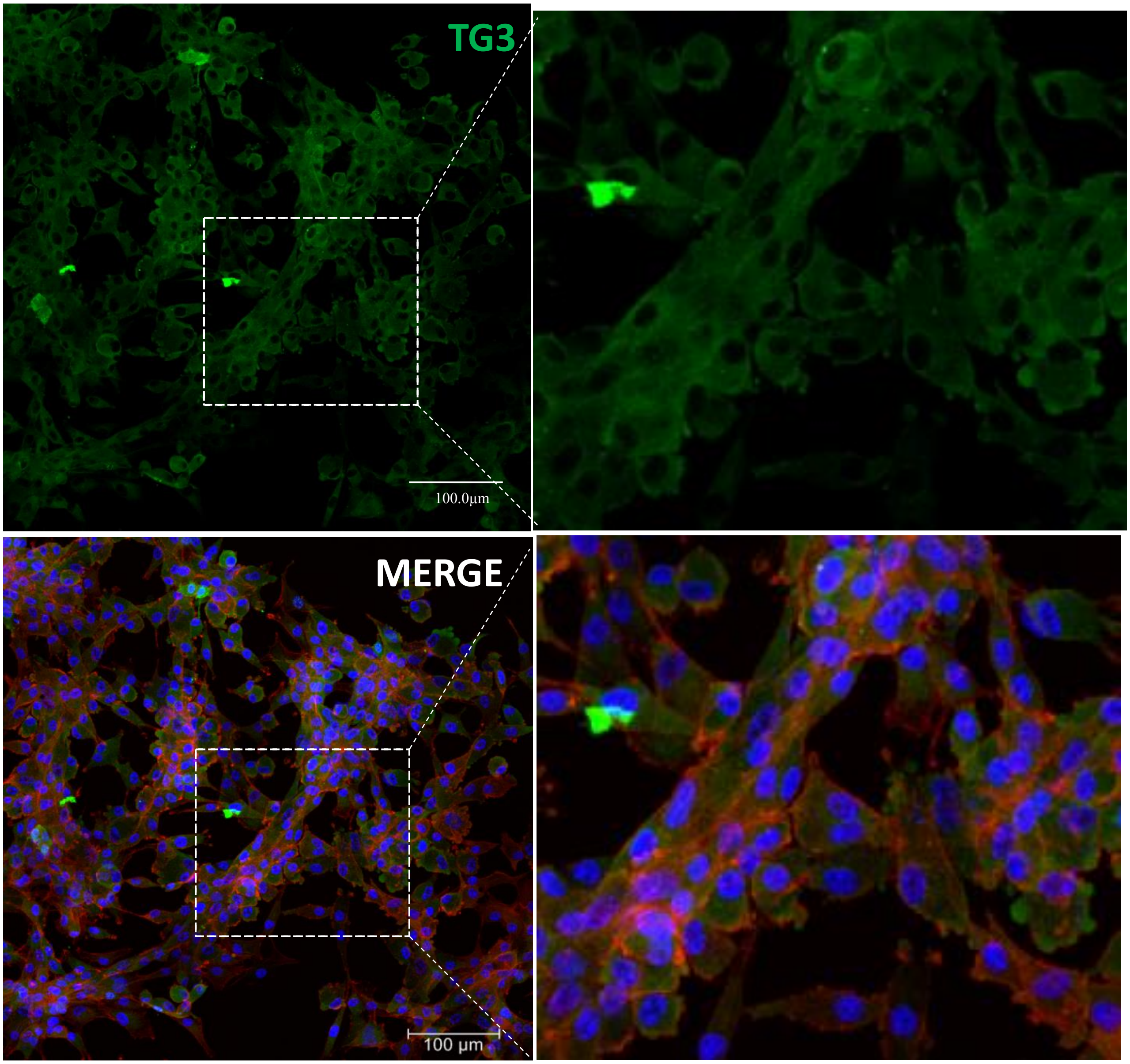

FigS3Bb
The daughter DPSCs that finished mitotic division display tau in the reconstituting nuclei; the nucleus of an interphase cell does not contain the epitope in active state. Counterstaining of nuclei with TOP-RO 3 (blue) and F-Actin by phalloidin confirms the cell's integrity

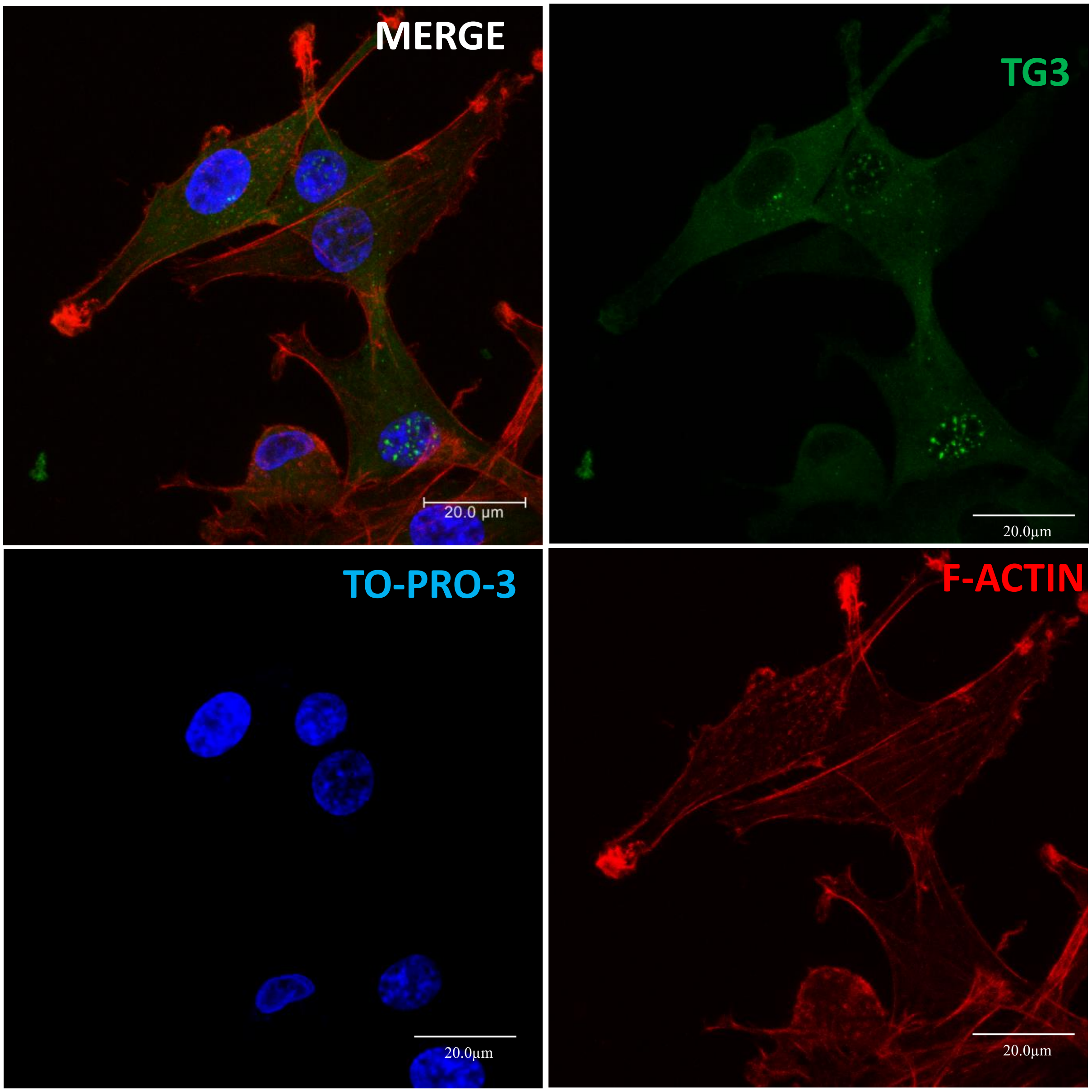

Highlighting of tau in centrioles

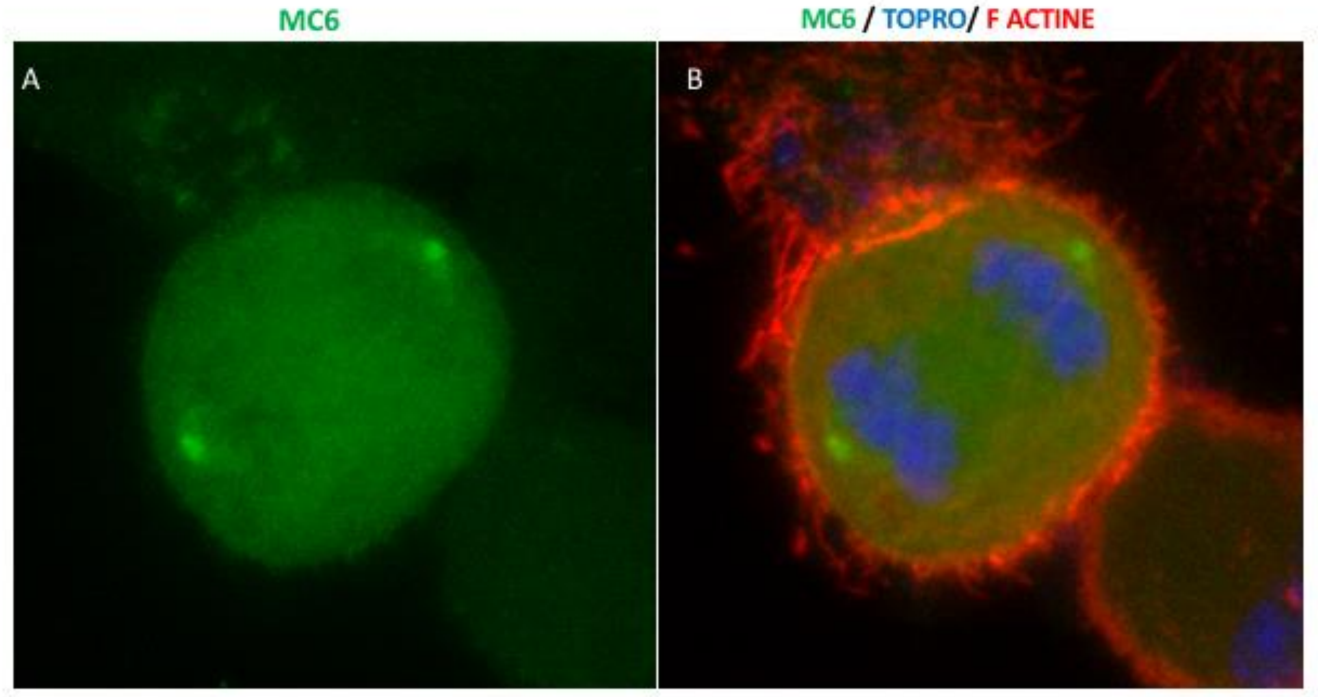

Neuronal propensity of DPSCs

DPSCs
Phase contrast

Neural markers
Flow cytometry

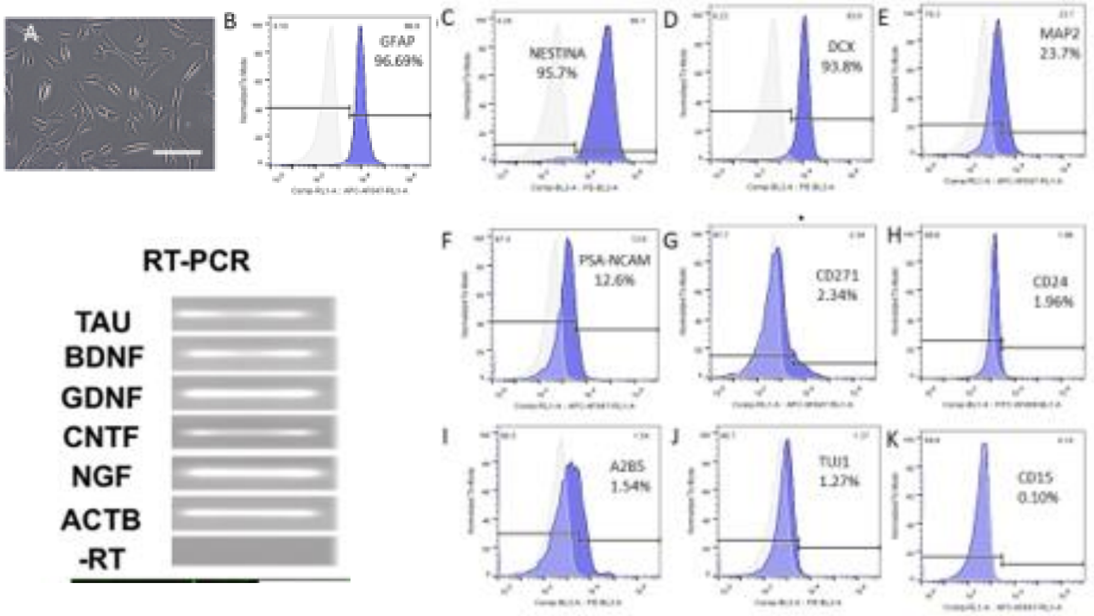

Supplement: Supplementary file 1 [file Data_Sheet_1.pdf]
